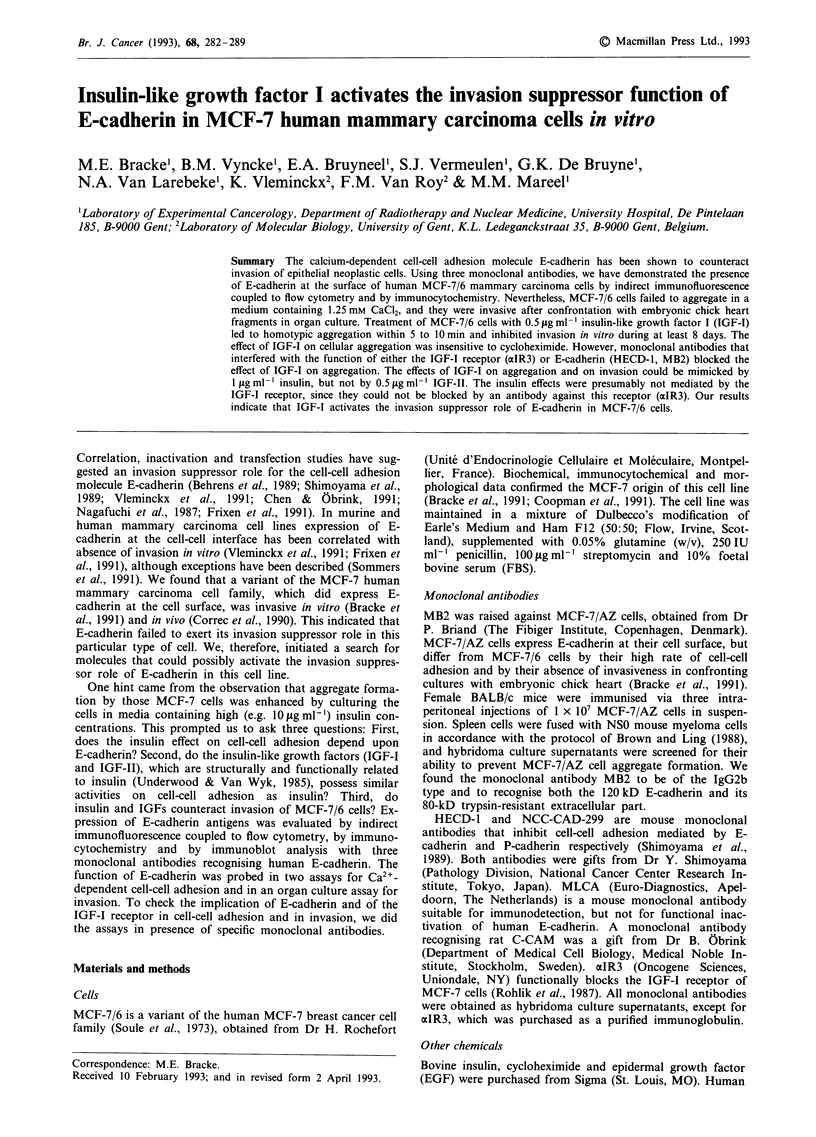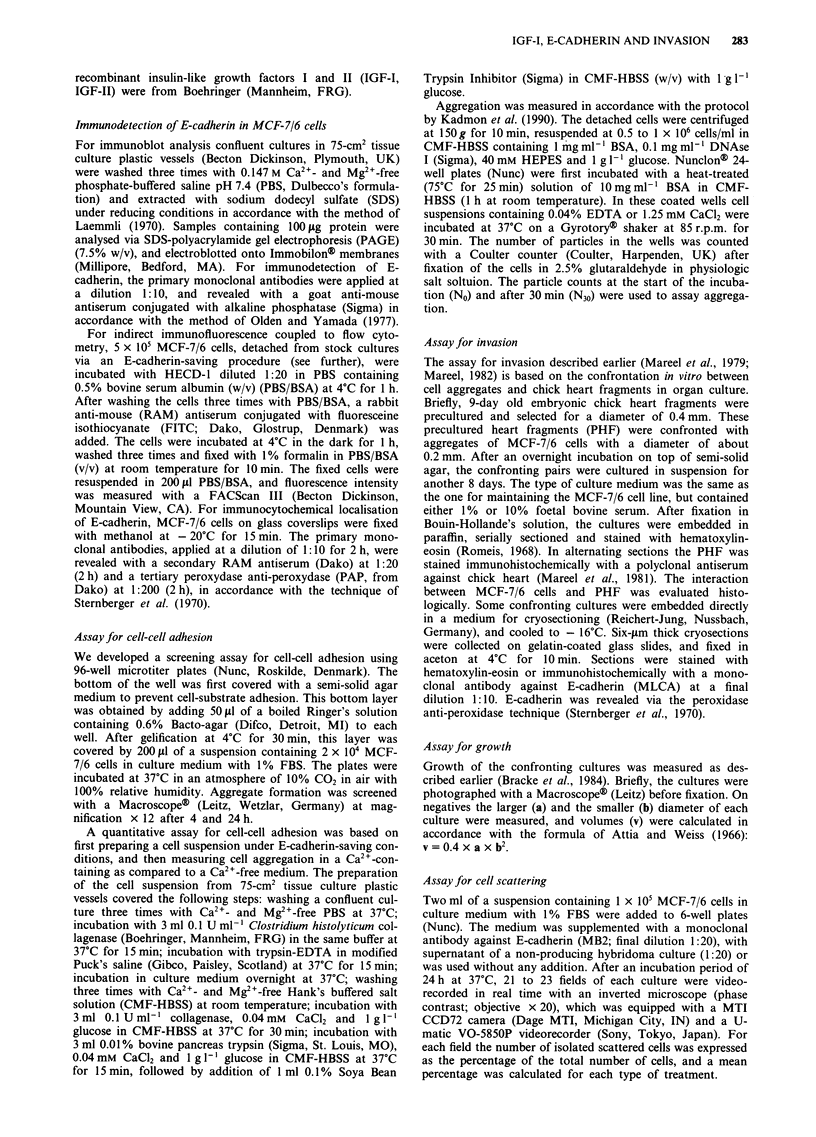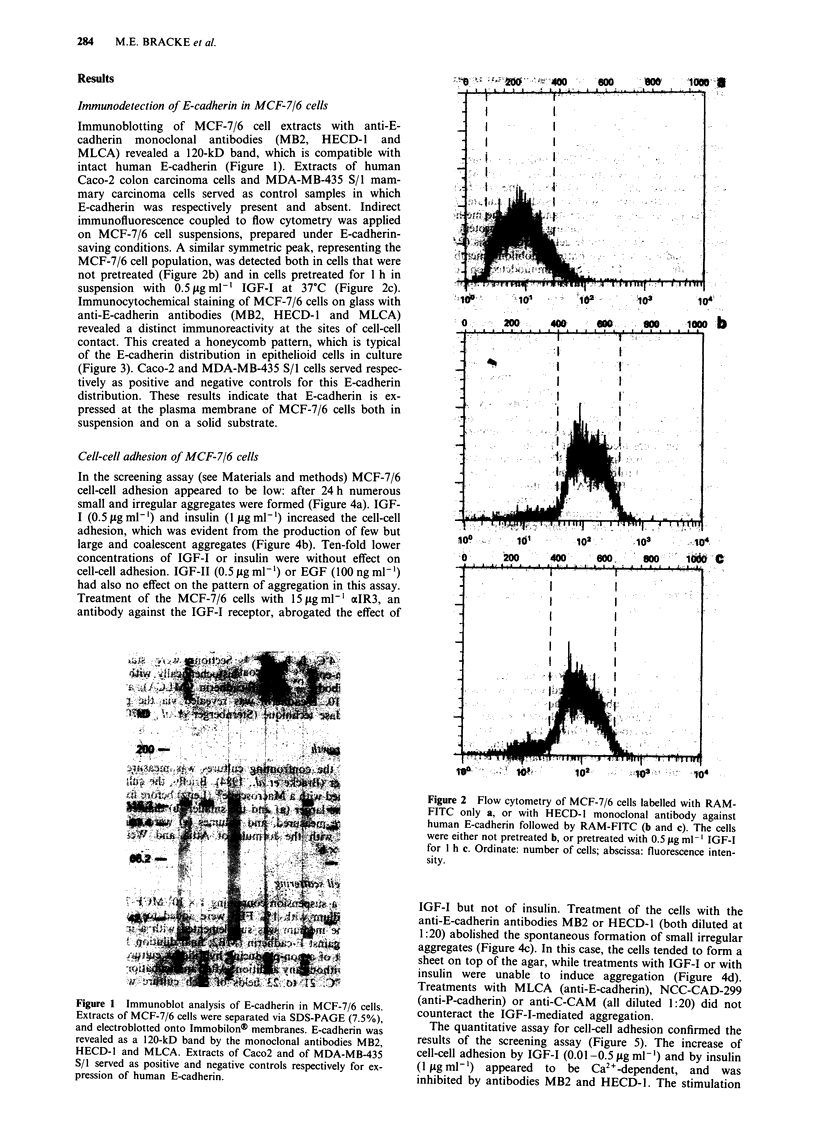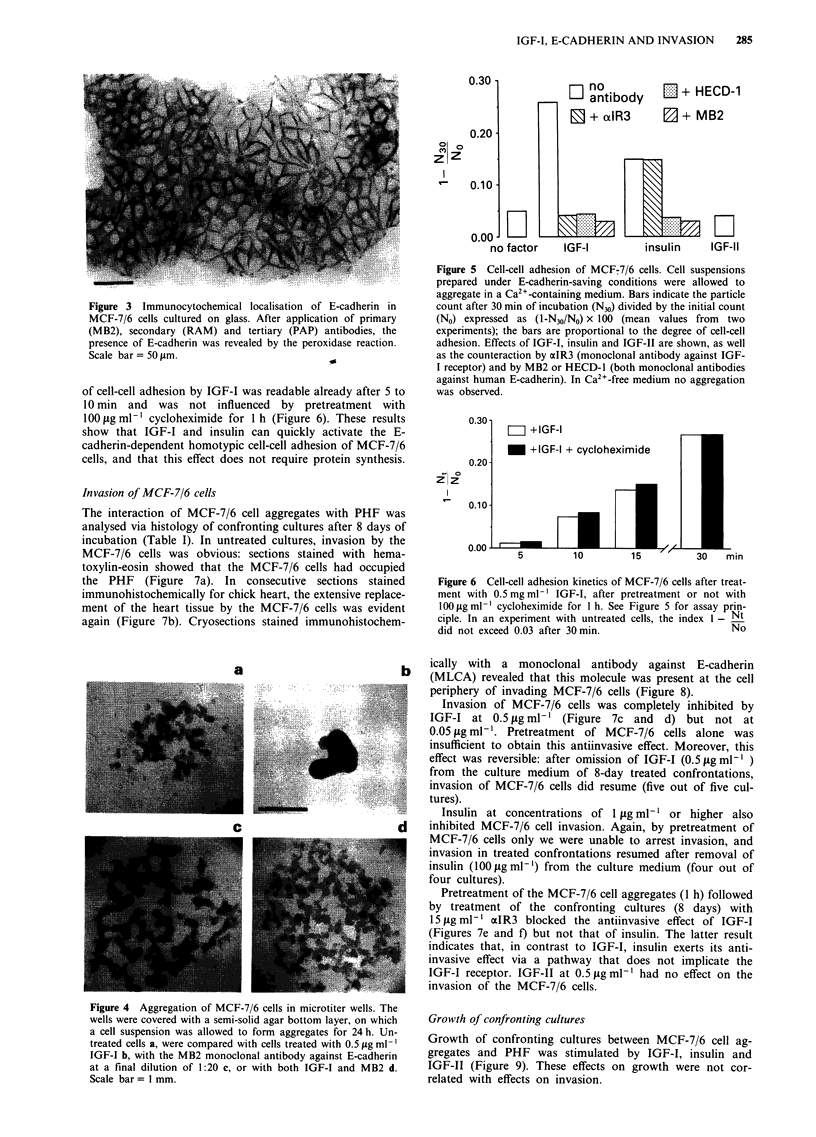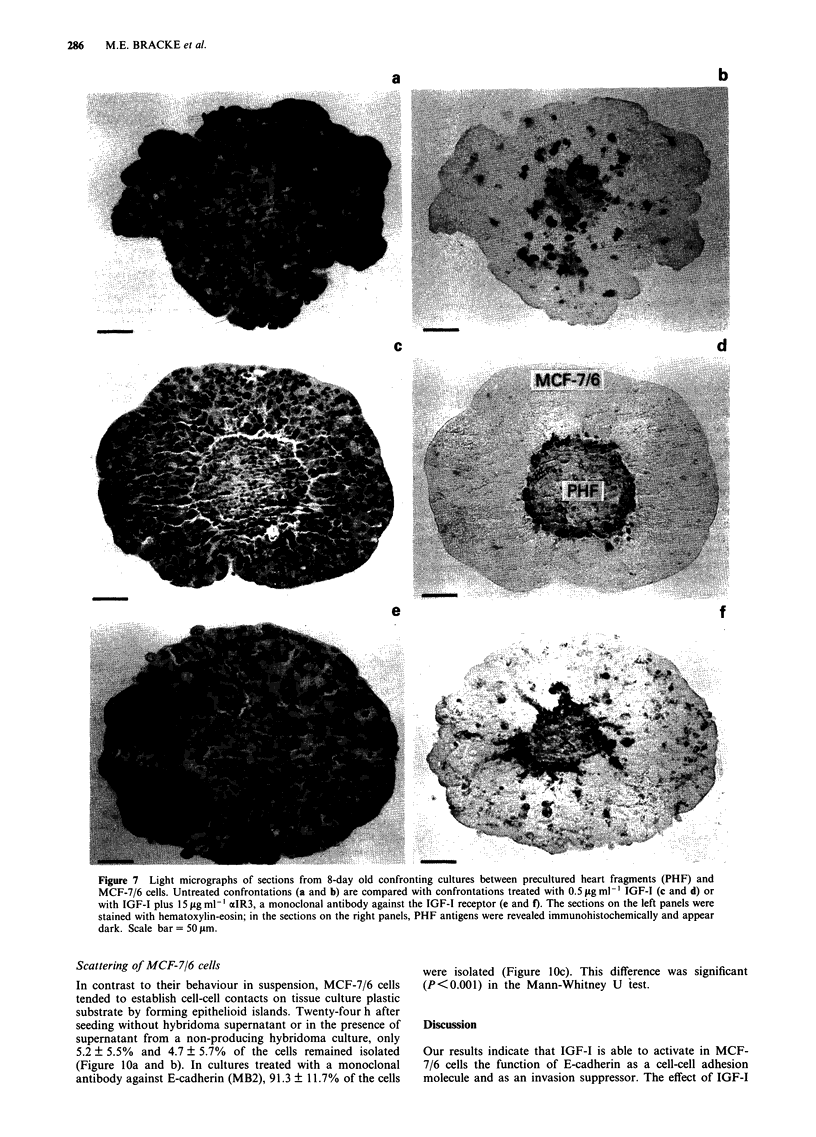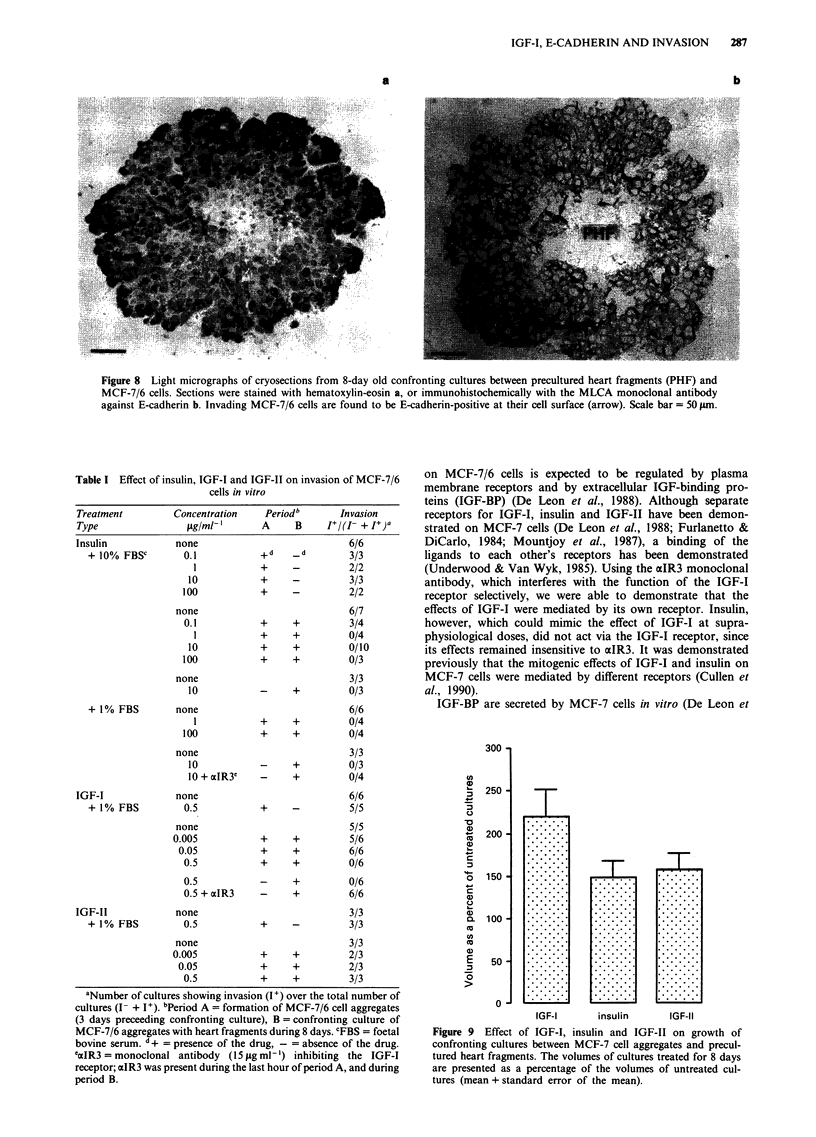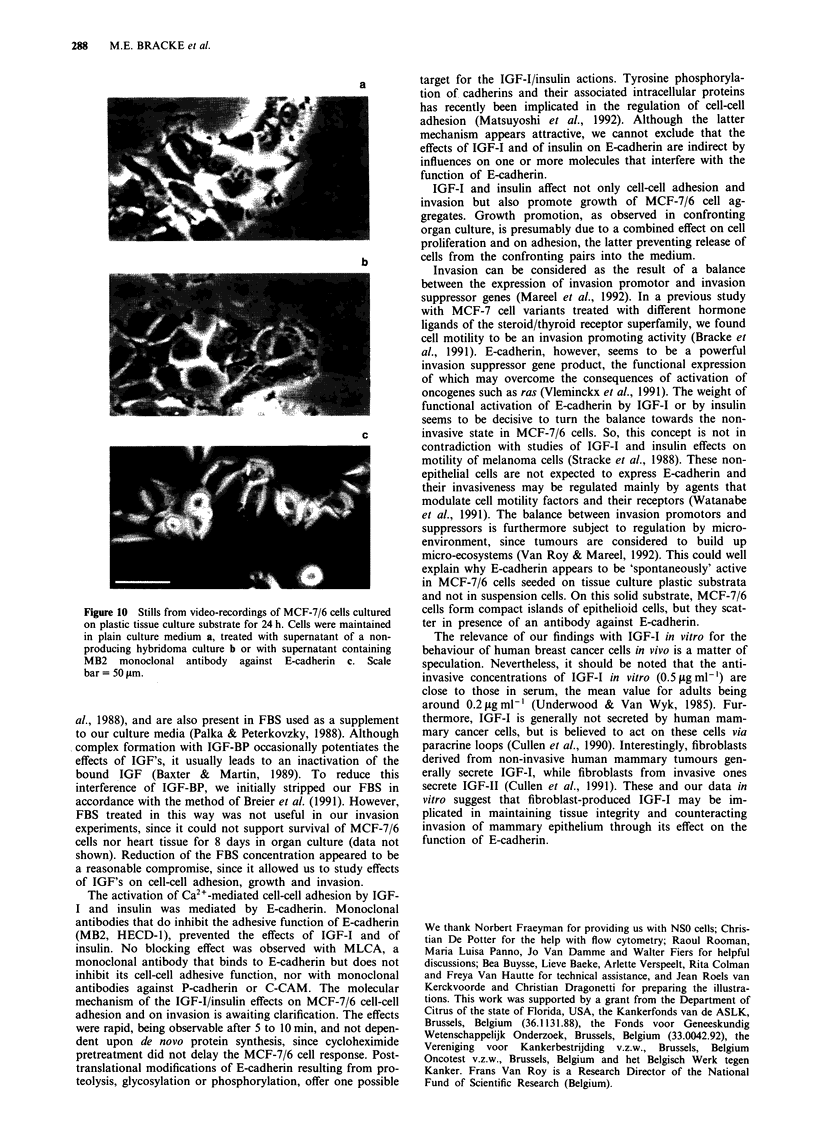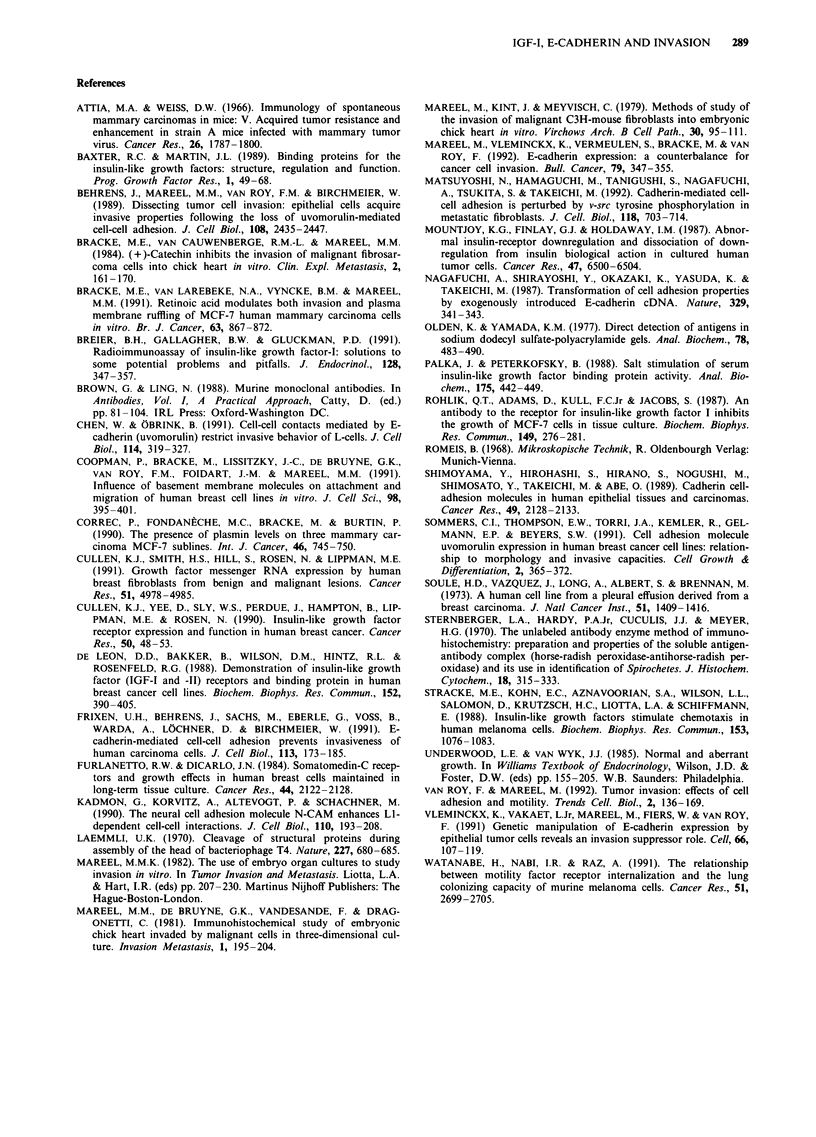# Insulin-like growth factor I activates the invasion suppressor function of E-cadherin in MCF-7 human mammary carcinoma cells in vitro.

**DOI:** 10.1038/bjc.1993.329

**Published:** 1993-08

**Authors:** M. E. Bracke, B. M. Vyncke, E. A. Bruyneel, S. J. Vermeulen, G. K. De Bruyne, N. A. Van Larebeke, K. Vleminckx, F. M. Van Roy, M. M. Mareel

**Affiliations:** Department of Radiotherapy and Nuclear Medicine, University Hospital, Gent, Belgium.

## Abstract

**Images:**


Br. J. Cancer (1993), 68, 282-289 © Macmillan Press Ltd., 1993

### Insulin-like growth factor I activates the invasion suppressor function of E-cadherin in MCF-7 human mammary carcinoma cells *in vitro*

M.E. Bracke[1], B.M. Vyncke[1], E.A. Bruyneel[1], S.J. Vermeulen[1], G.K. De Bruyne[1], N.A. Van Larebeke[1], K. Vleminckx[2], F.M. Van Roy[2] & M.M. Mareel[1]

[1]*Laboratory of Experimental Cancerology, Department of Radiotherapy and Nuclear Medicine, University Hospital, De Pintelaan 185, B-9000 Gent;* [2]*Laboratory of Molecular Biology, University of Gent, K.L. Ledeganckstraat 35, B-9000 Gent, Belgium.*

**Summary** The calcium-dependent cell-cell adhesion molecule E-cadherin has been shown to counteract invasion of epithelial neoplastic cells. Using three monoclonal antibodies, we have demonstrated the presence of E-cadherin at the surface of human MCF-7/6 mammary carcinoma cells by indirect immunofluorescence coupled to flow cytometry and by immunocytochemistry. Nevertheless, MCF-7/6 cells failed to aggregate in a medium containing 1.25 mM $CaCl_2$, and they were invasive after confrontation with embryonic chick heart fragments in organ culture. Treatment of MCF-7/6 cells with $0.5\,\mu g\,ml^{-1}$ insulin-like growth factor I (IGF-I) led to homotypic aggregation within 5 to 10 min and inhibited invasion *in vitro* during at least 8 days. The effect of IGF-I on cellular aggregation was insensitive to cycloheximide. However, monoclonal antibodies that interfered with the function of either the IGF-I receptor (αIR3) or E-cadherin (HECD-1, MB2) blocked the effect of IGF-I on aggregation. The effects of IGF-I on aggregation and on invasion could be mimicked by $1\,\mu g\,ml^{-1}$ insulin, but not by $0.5\,\mu g\,ml^{-1}$ IGF-II. The insulin effects were presumably not mediated by the IGF-I receptor, since they could not be blocked by an antibody against this receptor (αIR3). Our results indicate that IGF-I activates the invasion suppressor role of E-cadherin in MCF-7/6 cells.

Correlation, inactivation and transfection studies have suggested an invasion suppressor role for the cell-cell adhesion molecule E-cadherin (Behrens *et al.*, 1989; Shimoyama *et al.*, 1989; Vleminckx *et al.*, 1991; Chen & Öbrink, 1991; Nagafuchi *et al.*, 1987; Frixen *et al.*, 1991). In murine and human mammary carcinoma cell lines expression of E-cadherin at the cell-cell interface has been correlated with absence of invasion *in vitro* (Vleminckx *et al.*, 1991; Frixen *et al.*, 1991), although exceptions have been described (Sommers *et al.*, 1991). We found that a variant of the MCF-7 human mammary carcinoma cell family, which did express E-cadherin at the cell surface, was invasive *in vitro* (Bracke *et al.*, 1991) and *in vivo* (Correc *et al.*, 1990). This indicated that E-cadherin failed to exert its invasion suppressor role in this particular type of cell. We, therefore, initiated a search for molecules that could possibly activate the invasion suppressor role of E-cadherin in this cell line.

One hint came from the observation that aggregate formation by those MCF-7 cells was enhanced by culturing the cells in media containing high (e.g. $10\,\mu g\,ml^{-1}$) insulin concentrations. This prompted us to ask three questions: First, does the insulin effect on cell-cell adhesion depend upon E-cadherin? Second, do the insulin-like growth factors (IGF-I and IGF-II), which are structurally and functionally related to insulin (Underwood & Van Wyk, 1985), possess similar activities on cell-cell adhesion as insulin? Third, do insulin and IGFs counteract invasion of MCF-7/6 cells? Expression of E-cadherin antigens was evaluated by indirect immunofluorescence coupled to flow cytometry, by immunocytochemistry and by immunoblot analysis with three monoclonal antibodies recognising human E-cadherin. The function of E-cadherin was probed in two assays for $Ca^{2+}$-dependent cell-cell adhesion and in an organ culture assay for invasion. To check the implication of E-cadherin and of the IGF-I receptor in cell-cell adhesion and in invasion, we did the assays in presence of specific monoclonal antibodies.

### Materials and methods

### *Cells*

MCF-7/6 is a variant of the human MCF-7 breast cancer cell family (Soule *et al.*, 1973), obtained from Dr H. Rochefort (Unité d'Endocrinologie Cellulaire et Moléculaire, Montpellier, France). Biochemical, immunocytochemical and morphological data confirmed the MCF-7 origin of this cell line (Bracke *et al.*, 1991; Coopman *et al.*, 1991). The cell line was maintained in a mixture of Dulbecco's modification of Earle's Medium and Ham F12 (50:50; Flow, Irvine, Scotland), supplemented with 0.05% glutamine (w/v), 250 IU $ml^{-1}$ penicillin, $100\,\mu g\,ml^{-1}$ streptomycin and 10% foetal bovine serum (FBS).

### *Monoclonal antibodies*

MB2 was raised against MCF-7/AZ cells, obtained from Dr P. Briand (The Fibiger Institute, Copenhagen, Denmark). MCF-7/AZ cells express E-cadherin at their cell surface, but differ from MCF-7/6 cells by their high rate of cell-cell adhesion and by their absence of invasiveness in confronting cultures with embryonic chick heart (Bracke *et al.*, 1991). Female BALB/c mice were immunised via three intraperitoneal injections of $1 \times 10^7$ MCF-7/AZ cells in suspension. Spleen cells were fused with NS0 mouse myeloma cells in accordance with the protocol of Brown and Ling (1988), and hybridoma culture supernatants were screened for their ability to prevent MCF-7/AZ cell aggregate formation. We found the monoclonal antibody MB2 to be of the IgG2b type and to recognise both the 120 kD E-cadherin and its 80-kD trypsin-resistant extracellular part.

HECD-1 and NCC-CAD-299 are mouse monoclonal antibodies that inhibit cell-cell adhesion mediated by E-cadherin and P-cadherin respectively (Shimoyama *et al.*, 1989). Both antibodies were gifts from Dr Y. Shimoyama (Pathology Division, National Cancer Center Research Institute, Tokyo, Japan). MLCA (Euro-Diagnostics, Apeldoorn, The Netherlands) is a mouse monoclonal antibody suitable for immunodetection, but not for functional inactivation of human E-cadherin. A monoclonal antibody recognising rat C-CAM was a gift from Dr B. Öbrink (Department of Medical Cell Biology, Medical Noble Institute, Stockholm, Sweden). αIR3 (Oncogene Sciences, Uniondale, NY) functionally blocks the IGF-I receptor of MCF-7 cells (Rohlik *et al.*, 1987). All monoclonal antibodies were obtained as hybridoma culture supernatants, except for αIR3, which was purchased as a purified immunoglobulin.

### *Other chemicals*

Bovine insulin, cycloheximide and epidermal growth factor (EGF) were purchased from Sigma (St. Louis, MO). Human

Correspondence: M.E. Bracke.
Received 10 February 1993; and in revised form 2 April 1993.

recombinant insulin-like growth factors I and II (IGF-I, IGF-II) were from Boehringer (Mannheim, FRG).

*Immunodetection of E-cadherin in MCF-7/6 cells*

For immunoblot analysis confluent cultures in 75-cm$^2$ tissue culture plastic vessels (Becton Dickinson, Plymouth, UK) were washed three times with 0.147 M $Ca^{2+}$- and $Mg^{2+}$-free phosphate-buffered saline pH 7.4 (PBS, Dulbecco's formulation) and extracted with sodium dodecyl sulfate (SDS) under reducing conditions in accordance with the method of Laemmli (1970). Samples containing 100 µg protein were analysed via SDS-polyacrylamide gel electrophoresis (PAGE) (7.5% w/v), and electroblotted onto Immobilon® membranes (Millipore, Bedford, MA). For immunodetection of E-cadherin, the primary monoclonal antibodies were applied at a dilution 1:10, and revealed with a goat anti-mouse antiserum conjugated with alkaline phosphatase (Sigma) in accordance with the method of Olden and Yamada (1977).

For indirect immunofluorescence coupled to flow cytometry, $5 \times 10^5$ MCF-7/6 cells, detached from stock cultures via an E-cadherin-saving procedure (see further), were incubated with HECD-1 diluted 1:20 in PBS containing 0.5% bovine serum albumin (w/v) (PBS/BSA) at 4°C for 1 h. After washing the cells three times with PBS/BSA, a rabbit anti-mouse (RAM) antiserum conjugated with fluoresceine isothiocyanate (FITC; Dako, Glostrup, Denmark) was added. The cells were incubated at 4°C in the dark for 1 h, washed three times and fixed with 1% formalin in PBS/BSA (v/v) at room temperature for 10 min. The fixed cells were resuspended in 200 µl PBS/BSA, and fluorescence intensity was measured with a FACScan III (Becton Dickinson, Mountain View, CA). For immunocytochemical localisation of E-cadherin, MCF-7/6 cells on glass coverslips were fixed with methanol at − 20°C for 15 min. The primary monoclonal antibodies, applied at a dilution of 1:10 for 2 h, were revealed with a secondary RAM antiserum (Dako) at 1:20 (2 h) and a tertiary peroxydase anti-peroxydase (PAP, from Dako) at 1:200 (2 h), in accordance with the technique of Sternberger *et al.* (1970).

*Assay for cell-cell adhesion*

We developed a screening assay for cell-cell adhesion using 96-well microtiter plates (Nunc, Roskilde, Denmark). The bottom of the well was first covered with a semi-solid agar medium to prevent cell-substrate adhesion. This bottom layer was obtained by adding 50 µl of a boiled Ringer's solution containing 0.6% Bacto-agar (Difco, Detroit, MI) to each well. After gelification at 4°C for 30 min, this layer was covered by 200 µl of a suspension containing $2 \times 10^4$ MCF-7/6 cells in culture medium with 1% FBS. The plates were incubated at 37°C in an atmosphere of 10% $CO_2$ in air with 100% relative humidity. Aggregate formation was screened with a Macroscope® (Leitz, Wetzlar, Germany) at magnification × 12 after 4 and 24 h.

A quantitative assay for cell-cell adhesion was based on first preparing a cell suspension under E-cadherin-saving conditions, and then measuring cell aggregation in a $Ca^{2+}$-containing as compared to a $Ca^{2+}$-free medium. The preparation of the cell suspension from 75-cm$^2$ tissue culture plastic vessels covered the following steps: washing a confluent culture three times with $Ca^{2+}$- and $Mg^{2+}$-free PBS at 37°C; incubation with 3 ml 0.1 U ml$^{-1}$ *Clostridium histolyticum* collagenase (Boehringer, Mannheim, FRG) in the same buffer at 37°C for 15 min; incubation with trypsin-EDTA in modified Puck's saline (Gibco, Paisley, Scotland) at 37°C for 15 min; incubation in culture medium overnight at 37°C; washing three times with $Ca^{2+}$- and $Mg^{2+}$-free Hank's buffered salt solution (CMF-HBSS) at room temperature; incubation with 3 ml 0.1 U ml$^{-1}$ collagenase, 0.04 mM $CaCl_2$ and 1 g l$^{-1}$ glucose in CMF-HBSS at 37°C for 30 min; incubation with 3 ml 0.01% bovine pancreas trypsin (Sigma, St. Louis, MO), 0.04 mM $CaCl_2$ and 1 g l$^{-1}$ glucose in CMF-HBSS at 37°C for 15 min, followed by addition of 1 ml 0.1% Soya Bean Trypsin Inhibitor (Sigma) in CMF-HBSS (w/v) with 1 g l$^{-1}$ glucose.

Aggregation was measured in accordance with the protocol by Kadmon *et al.* (1990). The detached cells were centrifuged at 150 *g* for 10 min, resuspended at 0.5 to $1 \times 10^6$ cells/ml in CMF-HBSS containing 1 mg ml$^{-1}$ BSA, 0.1 mg ml$^{-1}$ DNAse I (Sigma), 40 mM HEPES and 1 g l$^{-1}$ glucose. Nunclon® 24-well plates (Nunc) were first incubated with a heat-treated (75°C for 25 min) solution of 10 mg ml$^{-1}$ BSA in CMF-HBSS (1 h at room temperature). In these coated wells cell suspensions containing 0.04% EDTA or 1.25 mM $CaCl_2$ were incubated at 37°C on a Gyrotory® shaker at 85 r.p.m. for 30 min. The number of particles in the wells was counted with a Coulter counter (Coulter, Harpenden, UK) after fixation of the cells in 2.5% glutaraldehyde in physiologic salt soltuion. The particle counts at the start of the incubation ($N_0$) and after 30 min ($N_{30}$) were used to assay aggregation.

*Assay for invasion*

The assay for invasion described earlier (Mareel *et al.*, 1979; Mareel, 1982) is based on the confrontation *in vitro* between cell aggregates and chick heart fragments in organ culture. Briefly, 9-day old embryonic chick heart fragments were precultured and selected for a diameter of 0.4 mm. These precultured heart fragments (PHF) were confronted with aggregates of MCF-7/6 cells with a diameter of about 0.2 mm. After an overnight incubation on top of semi-solid agar, the confronting pairs were cultured in suspension for another 8 days. The type of culture medium was the same as the one for maintaining the MCF-7/6 cell line, but contained either 1% or 10% foetal bovine serum. After fixation in Bouin-Hollande's solution, the cultures were embedded in paraffin, serially sectioned and stained with hematoxylin-eosin (Romeis, 1968). In alternating sections the PHF was stained immunohistochemically with a polyclonal antiserum against chick heart (Mareel *et al.*, 1981). The interaction between MCF-7/6 cells and PHF was evaluated histologically. Some confronting cultures were embedded directly in a medium for cryosectioning (Reichert-Jung, Nussbach, Germany), and cooled to − 16°C. Six-µm thick cryosections were collected on gelatin-coated glass slides, and fixed in aceton at 4°C for 10 min. Sections were stained with hematoxylin-eosin or immunohistochemically with a monoclonal antibody against E-cadherin (MLCA) at a final dilution 1:10. E-cadherin was revealed via the peroxidase anti-peroxidase technique (Sternberger *et al.*, 1970).

*Assay for growth*

Growth of the confronting cultures was measured as described earlier (Bracke *et al.*, 1984). Briefly, the cultures were photographed with a Macroscope® (Leitz) before fixation. On negatives the larger (**a**) and the smaller (**b**) diameter of each culture were measured, and volumes (**v**) were calculated in accordance with the formula of Attia and Weiss (1966): $\mathbf{v} = 0.4 \times \mathbf{a} \times \mathbf{b}^2$.

*Assay for cell scattering*

Two ml of a suspension containing $1 \times 10^5$ MCF-7/6 cells in culture medium with 1% FBS were added to 6-well plates (Nunc). The medium was supplemented with a monoclonal antibody against E-cadherin (MB2; final dilution 1:20), with supernatant of a non-producing hybridoma culture (1:20) or was used without any addition. After an incubation period of 24 h at 37°C, 21 to 23 fields of each culture were video-recorded in real time with an inverted microscope (phase contrast; objective × 20), which was equipped with a MTI CCD72 camera (Dage MTI, Michigan City, IN) and a U-matic VO-5850P videorecorder (Sony, Tokyo, Japan). For each field the number of isolated scattered cells was expressed as the percentage of the total number of cells, and a mean percentage was calculated for each type of treatment.

### Results

### *Immunodetection of E-cadherin in MCF-7/6 cells*

Immunoblotting of MCF-7/6 cell extracts with anti-E-cadherin monoclonal antibodies (MB2, HECD-1 and MLCA) revealed a 120-kD band, which is compatible with intact human E-cadherin (Figure 1). Extracts of human Caco-2 colon carcinoma cells and MDA-MB-435 S/1 mammary carcinoma cells served as control samples in which E-cadherin was respectively present and absent. Indirect immunofluorescence coupled to flow cytometry was applied on MCF-7/6 cell suspensions, prepared under E-cadherin-saving conditions. A similar symmetric peak, representing the MCF-7/6 cell population, was detected both in cells that were not pretreated (Figure 2b) and in cells pretreated for 1 h in suspension with 0.5 $\mu g\ ml^{-1}$ IGF-I at 37°C (Figure 2c). Immunocytochemical staining of MCF-7/6 cells on glass with anti-E-cadherin antibodies (MB2, HECD-1 and MLCA) revealed a distinct immunoreactivity at the sites of cell-cell contact. This created a honeycomb pattern, which is typical of the E-cadherin distribution in epithelioid cells in culture (Figure 3). Caco-2 and MDA-MB-435 S/1 cells served respectively as positive and negative controls for this E-cadherin distribution. These results indicate that E-cadherin is expressed at the plasma membrane of MCF-7/6 cells both in suspension and on a solid substrate.

### *Cell-cell adhesion of MCF-7/6 cells*

In the screening assay (see Materials and methods) MCF-7/6 cell-cell adhesion appeared to be low: after 24 h numerous small and irregular aggregates were formed (Figure 4a). IGF-I (0.5 $\mu g\ ml^{-1}$) and insulin (1 $\mu g\ ml^{-1}$) increased the cell-cell adhesion, which was evident from the production of few but large and coalescent aggregates (Figure 4b). Ten-fold lower concentrations of IGF-I or insulin were without effect on cell-cell adhesion. IGF-II (0.5 $\mu g\ ml^{-1}$) or EGF (100 $ng\ ml^{-1}$) had also no effect on the pattern of aggregation in this assay. Treatment of the MCF-7/6 cells with 15 $\mu g\ ml^{-1}$ αIR3, an antibody against the IGF-I receptor, abrogated the effect of IGF-I but not of insulin. Treatment of the cells with the anti-E-cadherin antibodies MB2 or HECD-1 (both diluted at 1:20) abolished the spontaneous formation of small irregular aggregates (Figure 4c). In this case, the cells tended to form a sheet on top of the agar, while treatments with IGF-I or with insulin were unable to induce aggregation (Figure 4d). Treatments with MLCA (anti-E-cadherin), NCC-CAD-299 (anti-P-cadherin) or anti-C-CAM (all diluted 1:20) did not counteract the IGF-I-mediated aggregation.

The quantitative assay for cell-cell adhesion confirmed the results of the screening assay (Figure 5). The increase of cell-cell adhesion by IGF-I (0.01–0.5 $\mu g\ ml^{-1}$) and by insulin (1 $\mu g\ ml^{-1}$) appeared to be $Ca^{2+}$-dependent, and was inhibited by antibodies MB2 and HECD-1. The stimulation

**Figure 1** Immunoblot analysis of E-cadherin in MCF-7/6 cells. Extracts of MCF-7/6 cells were separated via SDS-PAGE (7.5%), and electroblotted onto Immobilon® membranes. E-cadherin was revealed as a 120-kD band by the monoclonal antibodies MB2, HECD-1 and MLCA. Extracts of Caco2 and of MDA-MB-435 S/1 served as positive and negative controls respectively for expression of human E-cadherin.

**Figure 2** Flow cytometry of MCF-7/6 cells labelled with RAM-FITC only **a**, or with HECD-1 monoclonal antibody against human E-cadherin followed by RAM-FITC (**b** and **c**). The cells were either not pretreated **b**, or pretreated with 0.5 $\mu g\ ml^{-1}$ IGF-I for 1 h **c**. Ordinate: number of cells; abscissa: fluorescence intensity.

**Figure 3** Immunocytochemical localisation of E-cadherin in MCF-7/6 cells cultured on glass. After application of primary (MB2), secondary (RAM) and tertiary (PAP) antibodies, the presence of E-cadherin was revealed by the peroxidase reaction. Scale bar = 50 μm.

of cell-cell adhesion by IGF-I was readable already after 5 to 10 min and was not influenced by pretreatment with 100 μg $ml^{-1}$ cycloheximide for 1 h (Figure 6). These results show that IGF-I and insulin can quickly activate the E-cadherin-dependent homotypic cell-cell adhesion of MCF-7/6 cells, and that this effect does not require protein synthesis.

### *Invasion of MCF-7/6 cells*

The interaction of MCF-7/6 cell aggregates with PHF was analysed via histology of confronting cultures after 8 days of incubation (Table I). In untreated cultures, invasion by the MCF-7/6 cells was obvious: sections stained with hematoxylin-eosin showed that the MCF-7/6 cells had occupied the PHF (Figure 7a). In consecutive sections stained immunohistochemically for chick heart, the extensive replacement of the heart tissue by the MCF-7/6 cells was evident again (Figure 7b). Cryosections stained immunohistochemically with a monoclonal antibody against E-cadherin (MLCA) revealed that this molecule was present at the cell periphery of invading MCF-7/6 cells (Figure 8).

**Figure 4** Aggregation of MCF-7/6 cells in microtiter wells. The wells were covered with a semi-solid agar bottom layer, on which a cell suspension was allowed to form aggregates for 24 h. Untreated cells **a**, were compared with cells treated with 0.5 μg $ml^{-1}$ IGF-I **b**, with the MB2 monoclonal antibody against E-cadherin at a final dilution of 1:20 **c**, or with both IGF-I and MB2 **d**. Scale bar = 1 mm.

**Figure 5** Cell-cell adhesion of MCF-7/6 cells. Cell suspensions prepared under E-cadherin-saving conditions were allowed to aggregate in a $Ca^{2+}$-containing medium. Bars indicate the particle count after 30 min of incubation ($N_{30}$) divided by the initial count ($N_0$) expressed as ($1-N_{30}/N_0$) × 100 (mean values from two experiments); the bars are proportional to the degree of cell-cell adhesion. Effects of IGF-I, insulin and IGF-II are shown, as well as the counteraction by αIR3 (monoclonal antibody against IGF-I receptor) and by MB2 or HECD-1 (both monoclonal antibodies against human E-cadherin). In $Ca^{2+}$-free medium no aggregation was observed.

**Figure 6** Cell-cell adhesion kinetics of MCF-7/6 cells after treatment with 0.5 mg $ml^{-1}$ IGF-I, after pretreatment or not with 100 μg $ml^{-1}$ cycloheximide for 1 h. See Figure 5 for assay principle. In an experiment with untreated cells, the index $1-\frac{Nt}{No}$ did not exceed 0.03 after 30 min.

Invasion of MCF-7/6 cells was completely inhibited by IGF-I at 0.5 μg $ml^{-1}$ (Figure 7c and d) but not at 0.05 μg $ml^{-1}$. Pretreatment of MCF-7/6 cells alone was insufficient to obtain this antiinvasive effect. Moreover, this effect was reversible: after omission of IGF-I (0.5 μg $ml^{-1}$ ) from the culture medium of 8-day treated confrontations, invasion of MCF-7/6 cells did resume (five out of five cultures).

Insulin at concentrations of 1 μg $ml^{-1}$ or higher also inhibited MCF-7/6 cell invasion. Again, by pretreatment of MCF-7/6 cells only we were unable to arrest invasion, and invasion in treated confrontations resumed after removal of insulin (100 μg $ml^{-1}$) from the culture medium (four out of four cultures).

Pretreatment of the MCF-7/6 cell aggregates (1 h) followed by treatment of the confronting cultures (8 days) with 15 μg $ml^{-1}$ αIR3 blocked the antiinvasive effect of IGF-I (Figures 7e and f) but not that of insulin. The latter result indicates that, in contrast to IGF-I, insulin exerts its anti-invasive effect via a pathway that does not implicate the IGF-I receptor. IGF-II at 0.5 μg $ml^{-1}$ had no effect on the invasion of the MCF-7/6 cells.

### *Growth of confronting cultures*

Growth of confronting cultures between MCF-7/6 cell aggregates and PHF was stimulated by IGF-I, insulin and IGF-II (Figure 9). These effects on growth were not correlated with effects on invasion.

**Figure 7** Light micrographs of sections from 8-day old confronting cultures between precultured heart fragments (PHF) and MCF-7/6 cells. Untreated confrontations (**a** and **b**) are compared with confrontations treated with 0.5 $\mu g\ ml^{-1}$ IGF-I (**c** and **d**) or with IGF-I plus 15 $\mu g\ ml^{-1}$ αIR3, a monoclonal antibody against the IGF-I receptor (**e** and **f**). The sections on the left panels were stained with hematoxylin-eosin; in the sections on the right panels, PHF antigens were revealed immunohistochemically and appear dark. Scale bar = 50 $\mu m$.

*Scattering of MCF-7/6 cells*

In contrast to their behaviour in suspension, MCF-7/6 cells tended to establish cell-cell contacts on tissue culture plastic substrate by forming epithelioid islands. Twenty-four h after seeding without hybridoma supernatant or in the presence of supernatant from a non-producing hybridoma culture, only 5.2 ± 5.5% and 4.7 ± 5.7% of the cells remained isolated (Figure 10a and b). In cultures treated with a monoclonal antibody against E-cadherin (MB2), 91.3 ± 11.7% of the cells were isolated (Figure 10c). This difference was significant ($P < 0.001$) in the Mann-Whitney U test.

### Discussion

Our results indicate that IGF-I is able to activate in MCF-7/6 cells the function of E-cadherin as a cell-cell adhesion molecule and as an invasion suppressor. The effect of IGF-I

**Figure 8** Light micrographs of cryosections from 8-day old confronting cultures between precultured heart fragments (PHF) and MCF-7/6 cells. Sections were stained with hematoxylin-eosin **a**, or immunohistochemically with the MLCA monoclonal antibody against E-cadherin **b**. Invading MCF-7/6 cells are found to be E-cadherin-positive at their cell surface (arrow). Scale bar = 50 μm.

**Table I** Effect of insulin, IGF-I and IGF-II on invasion of MCF-7/6 cells *in vitro*

| *Treatment Type* | *Concentration* $\mu g/ml^{-1}$ | *Period*[b] A | B | *Invasion* $I^+/(I^- + I^+)$[a] |
|---|---|---|---|---|
| Insulin | none | | | 6/6 |
| + 10% FBS[c] | 0.1 | +[d] | −[d] | 3/3 |
| | 1 | + | − | 2/2 |
| | 10 | + | − | 3/3 |
| | 100 | + | − | 2/2 |
| | none | | | 6/7 |
| | 0.1 | + | + | 3/4 |
| | 1 | + | + | 0/4 |
| | 10 | + | + | 0/10 |
| | 100 | + | + | 0/3 |
| | none | | | 3/3 |
| | 10 | − | + | 0/3 |
| + 1% FBS | none | | | 6/6 |
| | 1 | + | + | 0/4 |
| | 100 | + | + | 0/4 |
| | none | | | 3/3 |
| | 10 | − | + | 0/3 |
| | 10 + αIR3[c] | − | + | 0/4 |
| IGF-I | none | | | 6/6 |
| + 1% FBS | 0.5 | + | − | 5/5 |
| | none | | | 5/5 |
| | 0.005 | + | + | 5/6 |
| | 0.05 | + | + | 6/6 |
| | 0.5 | + | + | 0/6 |
| | 0.5 | − | + | 0/6 |
| | 0.5 + αIR3 | − | + | 6/6 |
| IGF-II | none | | | 3/3 |
| + 1% FBS | 0.5 | + | − | 3/3 |
| | none | | | 3/3 |
| | 0.005 | + | + | 2/3 |
| | 0.05 | + | + | 2/3 |
| | 0.5 | + | + | 3/3 |

[a]Number of cultures showing invasion ($I^+$) over the total number of cultures ($I^- + I^+$). [b]Period A = formation of MCF-7/6 cell aggregates (3 days preceeding confronting culture), B = confronting culture of MCF-7/6 aggregates with heart fragments during 8 days. [c]FBS = foetal bovine serum. [d]+ = presence of the drug, − = absence of the drug. [e]αIR3 = monoclonal antibody (15 μg $ml^{-1}$) inhibiting the IGF-I receptor; αIR3 was present during the last hour of period A, and during period B.

on MCF-7/6 cells is expected to be regulated by plasma membrane receptors and by extracellular IGF-binding proteins (IGF-BP) (De Leon *et al.*, 1988). Although separate receptors for IGF-I, insulin and IGF-II have been demonstrated on MCF-7 cells (De Leon *et al.*, 1988; Furlanetto & DiCarlo, 1984; Mountjoy *et al.*, 1987), a binding of the ligands to each other's receptors has been demonstrated (Underwood & Van Wyk, 1985). Using the αIR3 monoclonal antibody, which interferes with the function of the IGF-I receptor selectively, we were able to demonstrate that the effects of IGF-I were mediated by its own receptor. Insulin, however, which could mimic the effect of IGF-I at supraphysiological doses, did not act via the IGF-I receptor, since its effects remained insensitive to αIR3. It was demonstrated previously that the mitogenic effects of IGF-I and insulin on MCF-7 cells were mediated by different receptors (Cullen *et al.*, 1990).

IGF-BP are secreted by MCF-7 cells *in vitro* (De Leon *et*

**Figure 9** Effect of IGF-I, insulin and IGF-II on growth of confronting cultures between MCF-7 cell aggregates and precultured heart fragments. The volumes of cultures treated for 8 days are presented as a percentage of the volumes of untreated cultures (mean + standard error of the mean).

**Figure 10** Stills from video-recordings of MCF-7/6 cells cultured on plastic tissue culture substrate for 24 h. Cells were maintained in plain culture medium **a**, treated with supernatant of a non-producing hybridoma culture **b** or with supernatant containing MB2 monoclonal antibody against E-cadherin **c**. Scale bar = 50 μm.

*al.*, 1988), and are also present in FBS used as a supplement to our culture media (Palka & Peterkovzky, 1988). Although complex formation with IGF-BP occasionally potentiates the effects of IGF's, it usually leads to an inactivation of the bound IGF (Baxter & Martin, 1989). To reduce this interference of IGF-BP, we initially stripped our FBS in accordance with the method of Breier *et al.* (1991). However, FBS treated in this way was not useful in our invasion experiments, since it could not support survival of MCF-7/6 cells nor heart tissue for 8 days in organ culture (data not shown). Reduction of the FBS concentration appeared to be a reasonable compromise, since it allowed us to study effects of IGF's on cell-cell adhesion, growth and invasion.

The activation of $Ca^{2+}$-mediated cell-cell adhesion by IGF-I and insulin was mediated by E-cadherin. Monoclonal antibodies that do inhibit the adhesive function of E-cadherin (MB2, HECD-1), prevented the effects of IGF-I and of insulin. No blocking effect was observed with MLCA, a monoclonal antibody that binds to E-cadherin but does not inhibit its cell-cell adhesive function, nor with monoclonal antibodies against P-cadherin or C-CAM. The molecular mechanism of the IGF-I/insulin effects on MCF-7/6 cell-cell adhesion and on invasion is awaiting clarification. The effects were rapid, being observable after 5 to 10 min, and not dependent upon *de novo* protein synthesis, since cycloheximide pretreatment did not delay the MCF-7/6 cell response. Post-translational modifications of E-cadherin resulting from proteolysis, glycosylation or phosphorylation, offer one possible target for the IGF-I/insulin actions. Tyrosine phosphorylation of cadherins and their associated intracellular proteins has recently been implicated in the regulation of cell-cell adhesion (Matsuyoshi *et al.*, 1992). Although the latter mechanism appears attractive, we cannot exclude that the effects of IGF-I and of insulin on E-cadherin are indirect by influences on one or more molecules that interfere with the function of E-cadherin.

IGF-I and insulin affect not only cell-cell adhesion and invasion but also promote growth of MCF-7/6 cell aggregates. Growth promotion, as observed in confronting organ culture, is presumably due to a combined effect on cell proliferation and on adhesion, the latter preventing release of cells from the confronting pairs into the medium.

Invasion can be considered as the result of a balance between the expression of invasion promotor and invasion suppressor genes (Mareel *et al.*, 1992). In a previous study with MCF-7 cell variants treated with different hormone ligands of the steroid/thyroid receptor superfamily, we found cell motility to be an invasion promoting activity (Bracke *et al.*, 1991). E-cadherin, however, seems to be a powerful invasion suppressor gene product, the functional expression of which may overcome the consequences of activation of oncogenes such as *ras* (Vleminckx *et al.*, 1991). The weight of functional activation of E-cadherin by IGF-I or by insulin seems to be decisive to turn the balance towards the non-invasive state in MCF-7/6 cells. So, this concept is not in contradiction with studies of IGF-I and insulin effects on motility of melanoma cells (Stracke *et al.*, 1988). These non-epithelial cells are not expected to express E-cadherin and their invasiveness may be regulated mainly by agents that modulate cell motility factors and their receptors (Watanabe *et al.*, 1991). The balance between invasion promotors and suppressors is furthermore subject to regulation by micro-environment, since tumours are considered to build up micro-ecosystems (Van Roy & Mareel, 1992). This could well explain why E-cadherin appears to be 'spontaneously' active in MCF-7/6 cells seeded on tissue culture plastic substrata and not in suspension cells. On this solid substrate, MCF-7/6 cells form compact islands of epithelioid cells, but they scatter in presence of an antibody against E-cadherin.

The relevance of our findings with IGF-I *in vitro* for the behaviour of human breast cancer cells *in vivo* is a matter of speculation. Nevertheless, it should be noted that the anti-invasive concentrations of IGF-I *in vitro* ($0.5\,\mu g\,ml^{-1}$) are close to those in serum, the mean value for adults being around $0.2\,\mu g\,ml^{-1}$ (Underwood & Van Wyk, 1985). Furthermore, IGF-I is generally not secreted by human mammary cancer cells, but is believed to act on these cells *via* paracrine loops (Cullen *et al.*, 1990). Interestingly, fibroblasts derived from non-invasive human mammary tumours generally secrete IGF-I, while fibroblasts from invasive ones secrete IGF-II (Cullen *et al.*, 1991). These and our data *in vitro* suggest that fibroblast-produced IGF-I may be implicated in maintaining tissue integrity and counteracting invasion of mammary epithelium through its effect on the function of E-cadherin.

We thank Norbert Fraeyman for providing us with NSO cells; Christian De Potter for the help with flow cytometry; Raoul Rooman, Maria Luisa Panno, Jo Van Damme and Walter Fiers for helpful discussions; Bea Buysse, Lieve Baeke, Arlette Verspeelt, Rita Colman and Freya Van Hautte for technical assistance, and Jean Roels van Kerckvoorde and Christian Dragonetti for preparing the illustrations. This work was supported by a grant from the Department of Citrus of the state of Florida, USA, the Kankerfonds van de ASLK, Brussels, Belgium (36.1131.88), the Fonds voor Geneeskundig Wetenschappelijk Onderzoek, Brussels, Belgium (33.0042.92), the Vereniging voor Kankerbestrijding v.z.w., Brussels, Belgium Oncotest v.z.w., Brussels, Belgium and het Belgisch Werk tegen Kanker. Frans Van Roy is a Research Director of the National Fund of Scientific Research (Belgium).

### References

ATTIA, M.A. & WEISS, D.W. (1966). Immunology of spontaneous mammary carcinomas in mice: V. Acquired tumor resistance and enhancement in strain A mice infected with mammary tumor virus. *Cancer Res.*, **26**, 1787–1800.

BAXTER, R.C. & MARTIN, J.L. (1989). Binding proteins for the insulin-like growth factors: structure, regulation and function. *Prog. Growth Factor Res.*, **1**, 49–68.

BEHRENS, J., MAREEL, M.M., VAN ROY, F.M. & BIRCHMEIER, W. (1989). Dissecting tumor cell invasion: epithelial cells acquire invasive properties following the loss of uvomorulin-mediated cell-cell adhesion. *J. Cell Biol.*, **108**, 2435–2447.

BRACKE, M.E., VAN CAUWENBERGE, R.M.-L. & MAREEL, M.M. (1984). (+)-Catechin inhibits the invasion of malignant fibrosarcoma cells into chick heart *in vitro*. *Clin. Expl. Metastasis*, **2**, 161–170.

BRACKE, M.E., VAN LAREBEKE, N.A., VYNCKE, B.M. & MAREEL, M.M. (1991). Retinoic acid modulates both invasion and plasma membrane ruffling of MCF-7 human mammary carcinoma cells *in vitro*. *Br. J. Cancer*, **63**, 867–872.

BREIER, B.H., GALLAGHER, B.W. & GLUCKMAN, P.D. (1991). Radioimmunoassay of insulin-like growth factor-I: solutions to some potential problems and pitfalls. *J. Endocrinol.*, **128**, 347–357.

BROWN, G. & LING, N. (1988). Murine monoclonal antibodies. In *Antibodies, Vol. I, A Practical Approach*, Catty, D. (ed.) pp. 81–104. IRL Press: Oxford-Washington DC.

CHEN, W. & ÖBRINK, B. (1991). Cell-cell contacts mediated by E-cadherin (uvomorulin) restrict invasive behavior of L-cells. *J. Cell Biol.*, **114**, 319–327.

COOPMAN, P., BRACKE, M., LISSITZKY, J.-C., DE BRUYNE, G.K., VAN ROY, F.M., FOIDART, J.-M. & MAREEL, M.M. (1991). Influence of basement membrane molecules on attachment and migration of human breast cell lines *in vitro*. *J. Cell Sci.*, **98**, 395–401.

CORREC, P., FONDANÈCHE, M.C., BRACKE, M. & BURTIN, P. (1990). The presence of plasmin levels on three mammary carcinoma MCF-7 sublines. *Int. J. Cancer*, **46**, 745–750.

CULLEN, K.J., SMITH, H.S., HILL, S., ROSEN, N. & LIPPMAN, M.E. (1991). Growth factor messenger RNA expression by human breast fibroblasts from benign and malignant lesions. *Cancer Res.*, **51**, 4978–4985.

CULLEN, K.J., YEE, D., SLY, W.S., PERDUE, J., HAMPTON, B., LIPPMAN, M.E. & ROSEN, N. (1990). Insulin-like growth factor receptor expression and function in human breast cancer. *Cancer Res.*, **50**, 48–53.

DE LEON, D.D., BAKKER, B., WILSON, D.M., HINTZ, R.L. & ROSENFELD, R.G. (1988). Demonstration of insulin-like growth factor (IGF-I and -II) receptors and binding protein in human breast cancer cell lines. *Biochem. Biophys. Res. Commun.*, **152**, 390–405.

FRIXEN, U.H., BEHRENS, J., SACHS, M., EBERLE, G., VOSS, B., WARDA, A., LÖCHNER, D. & BIRCHMEIER, W. (1991). E-cadherin-mediated cell-cell adhesion prevents invasiveness of human carcinoma cells. *J. Cell Biol.*, **113**, 173–185.

FURLANETTO, R.W. & DICARLO, J.N. (1984). Somatomedin-C receptors and growth effects in human breast cells maintained in long-term tissue culture. *Cancer Res.*, **44**, 2122–2128.

KADMON, G., KORVITZ, A., ALTEVOGT, P. & SCHACHNER, M. (1990). The neural cell adhesion molecule N-CAM enhances L1-dependent cell-cell interactions. *J. Cell Biol.*, **110**, 193–208.

LAEMMLI, U.K. (1970). Cleavage of structural proteins during assembly of the head of bacteriophage T4. *Nature*, **227**, 680–685.

MAREEL, M.M.K. (1982). The use of embryo organ cultures to study invasion *in vitro*. In *Tumor Invasion and Metastasis*. Liotta, L.A. & Hart, I.R. (eds) pp. 207–230. Martinus Nijhoff Publishers: The Hague-Boston-London.

MAREEL, M.M., DE BRUYNE, G.K., VANDESANDE, F. & DRAGONETTI, C. (1981). Immunohistochemical study of embryonic chick heart invaded by malignant cells in three-dimensional culture. *Invasion Metastasis*, **1**, 195–204.

MAREEL, M., KINT, J. & MEYVISCH, C. (1979). Methods of study of the invasion of malignant C3H-mouse fibroblasts into embryonic chick heart *in vitro*. *Virchows Arch. B Cell Path.*, **30**, 95–111.

MAREEL, M., VLEMINCKX, K., VERMEULEN, S., BRACKE, M. & VAN ROY, F. (1992). E-cadherin expression: a counterbalance for cancer cell invasion. *Bull. Cancer*, **79**, 347–355.

MATSUYOSHI, N., HAMAGUCHI, M., TANIGUSHI, S., NAGAFUCHI, A., TSUKITA, S. & TAKEICHI, M. (1992). Cadherin-mediated cell-cell adhesion is perturbed by *v-src* tyrosine phosphorylation in metastatic fibroblasts. *J. Cell. Biol.*, **118**, 703–714.

MOUNTJOY, K.G., FINLAY, G.J. & HOLDAWAY, I.M. (1987). Abnormal insulin-receptor downregulation and dissociation of down-regulation from insulin biological action in cultured human tumor cells. *Cancer Res.*, **47**, 6500–6504.

NAGAFUCHI, A., SHIRAYOSHI, Y., OKAZAKI, K., YASUDA, K. & TAKEICHI, M. (1987). Transformation of cell adhesion properties by exogenously introduced E-cadherin cDNA. *Nature*, **329**, 341–343.

OLDEN, K. & YAMADA, K.M. (1977). Direct detection of antigens in sodium dodecyl sulfate-polyacrylamide gels. *Anal. Biochem.*, **78**, 483–490.

PALKA, J. & PETERKOFSKY, B. (1988). Salt stimulation of serum insulin-like growth factor binding protein activity. *Anal. Biochem.*, **175**, 442–449.

ROHLIK, Q.T., ADAMS, D., KULL, F.C.Jr & JACOBS, S. (1987). An antibody to the receptor for insulin-like growth factor I inhibits the growth of MCF-7 cells in tissue culture. *Biochem. Biophys. Res. Commun.*, **149**, 276–281.

ROMEIS, B. (1968). *Mikroskopische Technik*, R. Oldenbourgh Verlag: Munich-Vienna.

SHIMOYAMA, Y., HIROHASHI, S., HIRANO, S., NOGUSHI, M., SHIMOSATO, Y., TAKEICHI, M. & ABE, O. (1989). Cadherin cell-adhesion molecules in human epithelial tissues and carcinomas. *Cancer Res.*, **49**, 2128–2133.

SOMMERS, C.I., THOMPSON, E.W., TORRI, J.A., KEMLER, R., GELMANN, E.P. & BEYERS, S.W. (1991). Cell adhesion molecule uvomorulin expression in human breast cancer cell lines: relationship to morphology and invasive capacities. *Cell Growth & Differentiation*, **2**, 365–372.

SOULE, H.D., VAZQUEZ, J., LONG, A., ALBERT, S. & BRENNAN, M. (1973). A human cell line from a pleural effusion derived from a breast carcinoma. *J. Natl Cancer Inst.*, **51**, 1409–1416.

STERNBERGER, L.A., HARDY, P.A.Jr, CUCULIS, J.J. & MEYER, H.G. (1970). The unlabeled antibody enzyme method of immunohistochemistry: preparation and properties of the soluble antigen-antibody complex (horse-radish peroxidase-antihorse-radish peroxidase) and its use in identification of *Spirochetes*. *J. Histochem. Cytochem.*, **18**, 315–333.

STRACKE, M.E., KOHN, E.C., AZNAVOORIAN, S.A., WILSON, L.L., SALOMON, D., KRUTZSCH, H.C., LIOTTA, L.A. & SCHIFFMANN, E. (1988). Insulin-like growth factors stimulate chemotaxis in human melanoma cells. *Biochem. Biophys. Res. Commun.*, **153**, 1076–1083.

UNDERWOOD, L.E. & VAN WYK, J.J. (1985). Normal and aberrant growth. In *Williams Textbook of Endocrinology*, Wilson, J.D. & Foster, D.W. (eds) pp. 155–205. W.B. Saunders: Philadelphia.

VAN ROY, F. & MAREEL, M. (1992). Tumor invasion: effects of cell adhesion and motility. *Trends Cell. Biol.*, **2**, 136–169.

VLEMINCKX, K., VAKAET, L.Jr, MAREEL, M., FIERS, W. & VAN ROY, F. (1991) Genetic manipulation of E-cadherin expression by epithelial tumor cells reveals an invasion suppressor role. *Cell*, **66**, 107–119.

WATANABE, H., NABI, I.R. & RAZ, A. (1991). The relationship between motility factor receptor internalization and the lung colonizing capacity of murine melanoma cells. *Cancer Res.*, **51**, 2699–2705.